# Complete plastome sequence of *Croton laevigatus* Vahl (Euphorbiaceae): an endemic species in Hainan, China

**DOI:** 10.1080/23802359.2019.1704659

**Published:** 2020-01-09

**Authors:** Xin Liao, Hong-Xin Wang, Zhi-Xin Zhu, Hua-Feng Wang

**Affiliations:** Key Laboratory of Tropical Biological Resources of Ministry of Education, School of Life and Pharmaceutical Sciences, Hainan University, Haikou, China

**Keywords:** *Croton laevigatus*, plastome, phylogeny, genome structure, Euphorbiaceae

## Abstract

*Croton laevigatus* grows as an evergreen tree or shrub with 15 meters height. It is distributed in the dense or open forests of Hainan province, China. Here, we report and characterize the complete plastome of *C. laevigatus* in an effort to provide genomic resources useful for promoting its systematics research. The plastome of *C. laevigatus* is found to possess a total length 162,515 bp with the typical quadripartite structure of angiosperms, contains two Inverted Repeats (IRs) of 26,866 bp, a Large Single-Copy (LSC) region of 90,234 bp and a Small Single-Copy (SSC) region of 18,549 bp. The plastome contains 113 genes, consisting of 79 unique protein-coding genes, 30 unique tRNA genes and four unique rRNA genes. The overall A/T content in the plastome of *C. laevigatus* is 64.10%. The phylogenetic analysis indicated that *C. laevigatus* is close to *C. tiglium* within Euphorbiaceae in this study. The complete plastome sequence of *C. laevigatus* will provide a useful resource for the conservation genetics of this species as well as for the phylogenetic studies of Euphorbiaceae.

## Introduction

*Croton laevigatus* Vahl (Euphorbiaceae) is an evergreen tree or shrub.It can be up to height of 15 meters. As an endemic species in Hainan, it is distributed in dense or sparse forests. It grows in mountainous areas with an altitude of 100–600 m (Li and Hans-Joachim 2008). Consequently, the genetic and genomic information is needed to promote its systematics research and the development of conservation value of *C. laevigatus.* Here, we report and characterize the complete plastome of *C. laevigatus* (GenBank accession number: MN713923). This is the first report of a complete plastome for *C. laevigatus.*

In this study, *C. laevigatus* was sampled from Diaoluo Mountain (18.67°N, 109.88°E), which is a National Nature Reserve of Hainan, China. A voucher specimen (Wang et al., B7) and its DNA was deposited in the Herbarium of the Institute of Tropical Agriculture and Forestry (HUTB), Hainan University, Haikou, China.

The experiment procedure is as reported in Zhu et al. (2018). Around six Gb clean data were assembled against the plastome of *Ricinus communis* (NC_016736.1) (Maximo et al. 2011) using MITObim v1.8 (Hahn et al. 2013). The plastome was annotated using Geneious R8.0.2 (Biomatters Ltd., Auckland, New Zealand) against the plastome of *R. communis* (NC_016736.1). The annotation was corrected with DOGMA (Wyman et al. 2004).

The plastome of *C. laevigatus* is found to possess a total length 162,515 bp with the typical quadripartite structure of angiosperms, contains two Inverted Repeats (IRs) of 26,866 bp, a Large Single-Copy (LSC) region of 90,234 bp and a Small Single-Copy (SSC) region of 18,549 bp. The plastome contains 113 genes, consisting of 79 unique protein-coding genes, 30 unique tRNA genes and 4 unique rRNA genes. The overall A/T content in the plastome of *C. laevigatus* is 64.10%, which the corresponding value of the LSC, SSC and IR region were 66.40%, 70.70% and 58.10%, respectively.

**Figure 1.**
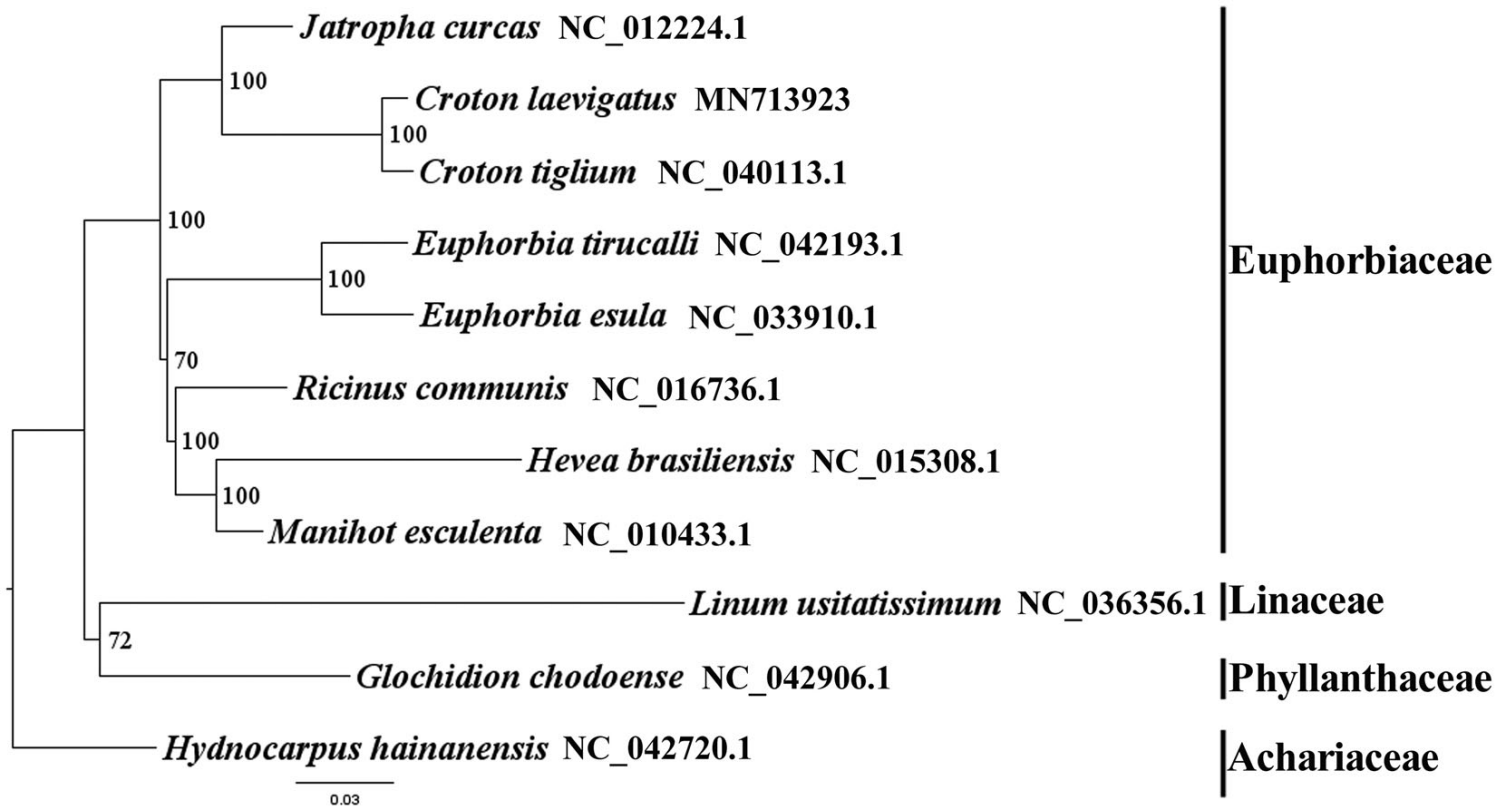
The best ML phylogeny recovered from 11 complete plastome sequences by RAxML. Accession numbers: *Croton laevigatus* MN713923, *Croton tiglium* NC_040113.1, *Ricinus communis* NC_016736.1, *Manihot esculenta* NC_010433.1, *Jatropha curcas* NC_012224.1, *Hevea brasiliensis* NC_015308.1, *Euphorbia tirucalli* NC_042193.1*, Euphorbia esula* NC_033910.1, *Glochidion chodoense* NC_042906.1, *Linum usitatissimum* NC_036356.1, *Hydnocarpus hainanensis* NC_042720.1.

We used RAxML (Stamatakis, 2006) with 1000 bootstraps under the GTRGAMMAI substitution model to reconstruct a maximum likelihood (ML) phylogeny of seven published complete plastomes of Euphorbiaceae, using three species of three families (Phyllanthaceae, Linaceae, Achariaceae) as outgroups. The phylogenetic analysis indicated that *C. laevigatus* is close to *C. tiglium* within Euphorbiaceae in this study (Figure 1). Most nodes in the plastome ML tree were strongly supported. The complete plastome sequence of *C. laevigatus* will provide a useful resource for the conservation genetics of this species as well as for the phylogenetic studies of Euphorbiaceae.
